# Cognitive Outcomes of Children Exposed to Selective Serotonin Reuptake Inhibitors Through Breast Milk

**DOI:** 10.1001/jamanetworkopen.2025.44989

**Published:** 2025-11-21

**Authors:** Essi Whaites Heinonen, Kelly Kao, Sarah N. Mattson, Christina D. Chambers

**Affiliations:** 1 Department of Pediatrics, Karolinska Institutet, Huddinge, Sweden; 2 Center for Better Beginnings, Department of Pediatrics, University of California, San Diego, La Jolla; 3 Center for Behavioral Teratology, San Diego State University, San Diego, California

## Abstract

**Importance:**

The cognitive performance of children prenatally exposed to selective serotonin reuptake inhibitors (SSRIs) has in some studies been shown to be reduced compared with that of unexposed children, potentially by factors associated with the underlying maternal disorder. However, cognitive performance following exposure to SSRIs through breastfeeding has not been previously studied.

**Objective:**

To determine whether the cognitive performance of children exposed to SSRIs both during pregnancy and breastfeeding differs from that of children with SSRI exposure limited to pregnancy.

**Design, Setting, and Participants:**

This prospective cohort study included the offspring of pregnant women enrolled in the MotherToBaby California cohort from May 8, 1989, to April 14, 2008. Children whose mothers were treated with SSRIs during pregnancy completed neurodevelopmental testing at 4 to 5 years of age (April 30, 1996, to August 12, 2012). Data were analyzed from January 10 to May 16, 2025.

**Exposures:**

Children exposed to SSRIs during breastfeeding were compared with breastfed children not exposed to SSRIs during breastfeeding and nonbreastfed children.

**Main Outcomes and Measures:**

Full-scale, verbal, and performance IQ were measured with the Wechsler Scales of Preschool and Primary Intelligence and compared using analysis of covariance adjusted for child sex, prematurity, and age at testing.

**Results:**

The sample included 97 mother-child dyads. Of the 97 children, 52 (53.6%) were female and 45 (46.4%) were male, with a mean (SD) age at testing of 4.9 (0.7) years. Of these, 22 children (22.7%) were exposed to SSRIs during breastfeeding; 37 (38.1%) were breastfed without SSRI exposure; and 38 (39.2%) were not breastfed. There were no significant differences in any adjusted mean IQ scores between breastfed children with and without postnatal SSRI exposure. Compared with nonbreastfed children, breastfed children exposed to SSRIs postnatally had significantly higher adjusted mean full-scale IQ scores (109.4 [95% CI, 104.5-114.4] vs 103.1 [95% CI, 99.3-106.9]; *P* = .046) and performance IQ scores (112.3 [95% CI, 106.7-118.0] vs 104.2 [95% CI, 99.9-108.5]; *P* = .03), but these differences were no longer significant after adjustment for factors related to the maternal mood during pregnancy.

**Conclusions and Relevance:**

In this cohort study including children prenatally exposed to SSRIs, additional exposure to SSRIs through breastfeeding was not associated with reduced IQ scores. Although measures of performance other than IQ were not evaluated, these findings suggest that breastfeeding can be encouraged during treatment with SSRIs.

## Key Points

**Question:**

Is maternal treatment with selective serotonin reuptake inhibitors (SSRIs) during breastfeeding associated with cognitive performance of the offspring?

**Findings:**

This cohort study of 97 children who were exposed to maternal treatment with SSRIs during pregnancy with or without exposure during breastfeeding underwent testing with the Wechsler Scales of Preschool and Primary Intelligence. Fully adjusted mean full-scale, verbal, and performance IQ scores were similar among breastfed children exposed to SSRIs, breastfed children not exposed to SSRIs, and nonbreastfed children.

**Meaning:**

These findings suggest that maternal treatment with SSRIs during breastfeeding does not affect the cognitive performance of the offspring.

## Teaser

This cohort study compares cognitive performance in preschool-aged children who were exposed to selective serotonin reuptake inhibitors (SSRIs) during pregnancy and breastfeeding, those who were breastfed but only exposed during pregnancy, and those who were not breastfed.

## Introduction

The cognitive development of children exposed to maternal treatment with selective serotonin reuptake inhibitors (SSRIs) during pregnancy has been studied extensively.^1,2,3,4,5^ Most previous studies have shown either no significant effects of prenatal SSRI exposure on the child development or effects that were attenuated or eliminated after adjustments for factors related to the underlying maternal disorder.^1,5^ However, to our knowledge, the association between child cognitive performance and exposure to SSRIs through breastfeeding has not been previously examined. This question is of clinical importance, as some breastfeeding mothers still avoid antidepressant use due to the effects treatment may pose for the breastfed infant.^6,7,8,9^

SSRIs are known to transfer to breast milk with maximum relative infant doses of 4% to 12%, with fluoxetine hydrochloride, citalopram, and escitalopram transferring to the highest degree.^10,11,12^ Although the level of drug exposure is lower through breastfeeding than through placental transfer, there is at least a theoretical risk that the highly lipophilic SSRIs ingested by the breastfed infant could negatively affect the infant’s brain maturation.^10,13,14,15,16,17^ However, breastfeeding itself is known to have numerous positive effects, including on the cognitive development of the child.^15,18,19^ These benefits may well outweigh any potential negative effects that exposure to SSRIs through breast milk may have. Exclusive breastfeeding for at least 6 months is recommended for all infants, and hence, it is essential to provide adequate safety data for the long-term development of children exposed to SSRIs through breastfeeding to address remaining avoidant behavior regarding breastfeeding among mothers treated with SSRIs.^6,7,20^

In this study, we compared the cognitive performance measured with the Wechsler Primary and Preschool Test of Intelligence (WPPSI) in preschool-aged children among those who were exposed to SSRIs during pregnancy and breastfeeding, those who were breastfed but only exposed during pregnancy, and those who were not breastfed. We hypothesized that the IQ scores of children exposed to SSRIs during breastfeeding would be comparable to the scores of children breastfed without postnatal SSRI exposure and somewhat higher than the scores of nonbreastfed children.

## Methods

### Design and Setting

This cohort study was a secondary analysis of data in the MotherToBaby California cohort that was open for enrollment for all English- and Spanish-speaking women in California. Women were eligible to enroll in the study if they did not have prior knowledge of the pregnancy outcome and agreed to the study protocol. The study design and methods have been described in detail elsewhere.^21,22,23^ Briefly, pregnant women were referred or self-referred into the single site research center during pregnancy and all liveborn infants were followed up to a minimum of 1 year after birth. Depending on their exposures, some participants, including those treated with SSRIs, were also invited to take part in face-to-face neurodevelopmental testing of the child when the child reached preschool age. Exposure, outcome, and covariate information during pregnancy and in the postpartum period were obtained through repeated telephone interviews and confirmed by medical records when available. Additional information was obtained at the time of neurodevelopmental testing.

**Figure 1.**
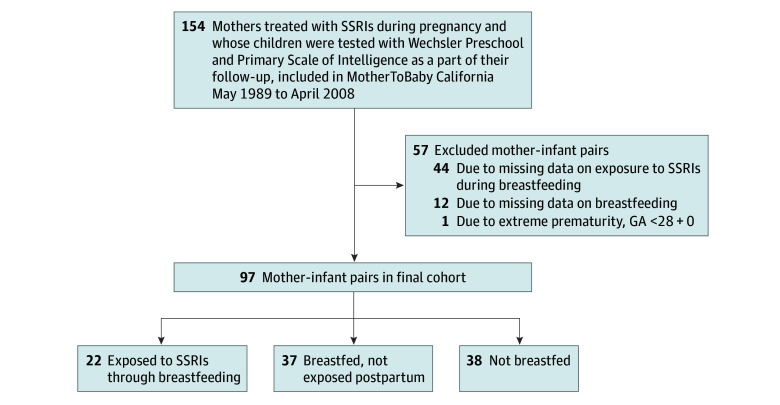
Flowchart of Study Cohort GA indicates gestational age (in weeks and days); SSRI, selective serotonin reuptake inhibitor.

For this analysis, eligible participants who enrolled in the MotherToBaby California cohort between May 18, 1989, and April 14, 2008, were treated with SSRIs at the time of enrollment in early pregnancy, and their children were tested with the WPPSI between April 30, 1996, and August 21, 2012. Mothers with missing information on breastfeeding and postnatal SSRI treatment as well as 1 extremely preterm child were excluded (Figure 1). The data were analyzed from January 10 to May 16, 2025. Institutional Review Board approval was obtained for the parent study from the University of California, San Diego, and San Diego State University. All participants provided written informed consent. The study adhered to the Strengthening the Reporting of Observational Studies in Epidemiology (STROBE) reporting guideline.

### Exposures

All mothers were treated with SSRIs during pregnancy at inclusion to the cohort. In this study, the children who were exposed to SSRIs through breastfeeding were compared with children whose mothers discontinued SSRI treatment before breastfeeding. Secondarily, the children exposed to SSRIs through breastfeeding were compared with the children who were not breastfed (Figure 1). Breastfeeding was defined as a known breastfeeding duration of at least 1 month, and hence, participants with breastfeeding data only from newborn records were excluded.

### Outcomes

The main outcomes were the composite scores of Full-Scale (FSIQ), Verbal (VIQ), and Performance IQ (PIQ) measured with the version of WPPSI available at the time of testing, the WPPSI Revised (WPPSI-R) or the WPPSI, Third Edition (WPPSI-III).^24,25^ The composite scores of the WPPSI tests are standardized to the age and sex of the child with a mean (SD) score of 100 (50) points. Secondary outcomes were scaled scores of 4 verbal and 8 nonverbal subtests of the WPPSI-R, with results from children tested with WPPSI-III included for the subtests that were similar to those in the WPPSI-R. These were also standardized to age and sex of the child with a mean (SD) of 10 (3) points. Included verbal subtests were Information, Comprehension, Vocabulary, and Similarities; nonverbal subtests were Picture Completion, Object Assembly, Block Design, Sentences, Arithmetic, Animal Pegs, Geometric Design, and Mazes. In a secondary analysis, we studied the correlations of the child IQ scores with maternal IQ and depressive symptoms.

### Covariates

Data on baseline characteristics and covariates were collected through maternal telephone interviews and are presented in Table 1. Due to both SSRI use and breastfeeding frequency varying by race and ethnicity, maternal self-reported race and ethnicity were collected at the interviews. Race and ethnicity were collected as 2 separate variables and combined for this analysis. The original categories for race in the interviews were American Indian or Alaska Native, Asian, Black, Native Hawaiian or Other Pacific Islander, White, other, refused, not asked, and unknown; categories for ethnicity included Hispanic or Latina and non-Hispanic or non-Latina. For the present study, we combined these categories as Hispanic or Latina (including White, other, unknown, and Hispanic or Latina), non-Hispanic Black (including Black and non-Hispanic or non-Latina), non-Hispanic White (including White and non-Hispanic or non-Latina), and other (including American Indian or Alaska Native, Asian, Native Hawaiian or Other Pacific Islander, other, and non-Hispanic or non-Latina). Socioeconomic status was measured with the Hollingshead 4-Factor Index representing maternal and paternal educational attainment and occupation scored from 1 to 5 and categorized into high (1-3) and low (4-5) socioeconomic status.^26^ Maternal depressive symptoms were estimated with the Center for Epidemiological Studies Depression Scale (CES-D) administered at the time of neurodevelopmental testing or later, as the CES-D was not initially a part of the testing procedure and was in some cases administered retroactively. In all cases, the mother was asked to rate their symptoms of depression both currently and retrospectively during the target pregnancy. A cutoff of 16 points was set for depressive symptoms, indicating a potential risk of clinical depression.^27^ In a subset of the cohort, maternal IQ was measured at the time of the child neurodevelopmental testing with the Wechsler Adult Scale of Intelligence. Covariates included in the adjusted analyses were child age at testing (continuous), child sex (binary), and prematurity (born at 34-36 weeks of gestation [binary]). In sensitivity analyses, maternal use of other psychotropic comedications (binary) and alcohol (binary) during pregnancy as well as the duration of treatment with SSRIs during pregnancy (continuous) and maternal CES-D scores during pregnancy (continuous) were included as covariates.

**Table 1.** Baseline Characteristics of Participating Mothers and Children

Characteristic	Participant group, No. (%)			*P* value^a^
	SSRI exposure during breastfeeding (n = 22)	Breastfed but no SSRI exposure (n = 37)	Not breastfed (n = 38)	
**Maternal characteristics**				
Maternal age at delivery, mean (SD), y	35.5 (5.4)	33.2 (5.3)	33.9 (6.0)	.18
Primipara	11 (50.0)	18 (48.6)	20 (52.6)	.94
Race and ethnicity^b^				
Hispanic or Latina	1 (4.5)	3 (8.1)	4 (10.8)	.98
Non-Hispanic White	20 (90.9)	32 (86.5)	32 (86.5)	
Other^c^	1 (4.5)	2 (5.4)	1 (2.7)	
Socioeconomic scale^d^				.32
1-3	20 (100)	31 (88.6)	32 (94.1)	
4-5	0	4 (11.4)	2 (5.9)	
Duration of treatment with SSRIs during pregnancy, mean (SD), wk	35.0 (11.2)	14.0 (12.4)	28.0 (14.8)	<.001
Indication for treatment with SSRIs				
Major depressive disorder	17 (77.3)	23 (62.2)	28 (73.7)	.15
Anxiety or panic disorder	1 (4.5)	4 (10.8)	2 (5.3)	
Depression with other psychiatric disorders^e^	1 (4.5)	0	4 (10.5)	
Other^f^	2 (9.1)	10 (27.0)	4 (10.5)	
Tobacco use during pregnancy	1 (4.5)	4 (10.8)	9 (27.3)	.07
Alcohol use during pregnancy	13 (59.1)	26 (70.3)	9 (27.3)	<.001
Psychotropic comedications during pregnancy	2 (9.1)	8 (21.6)	15 (39.5)	.03
CES-D score during pregnancy ≥16^g^	10 (50.0)	14 (43.8)	19 (59.4)	.45
CES-D score during pregnancy, mean (SD)	19.5 (15.3)	16.2 (13.3)	24.4 (16.5)	.09
CES-D score at the time of testing ≥16^h^	7 (36.8)	12 (38.7)	15 (46.9)	.72
CES-D score at the time of testing, mean (SD)	15.0 (14.3)	16.6 (13.4)	17.5 (13.9)	.68
Maternal IQ, mean (SD)	120.8 (7.5)	116.4 (5.4)	110.1 (14.7)	.14
Data availability for maternal IQ	6 (27.3)	11 (29.7)	14 (36.8)	.70
**Neonatal and breastfeeding characteristics**				
Gestational age at delivery, mean (SD), wk	39.5 (1.3)	39.4 (1.4)	39.2 (1.5)	.96
Late preterm infants	1 (4.5)	1 (2.7)	3 (7.9)	.84
Sex				
Female	10 (45.5)	21 (56.8)	21 (55.3)	.68
Male	12 (54.5)	16 (43.2)	17 (44.7)	
Admitted to neonatal intensive care	6 (27.3)	5 (13.5)	6 (15.8)	.12
Exclusive breastfeeding^i^	14 (87.5)	18 (72.0)	NA	.44
Breastfeeding duration at least, mean (SD), mo	14.1 (9.0)	5.0 (5.0)	NA	.002
Data availability for breastfeeding duration	11 (50.0)	16 (43.2)	NA	.79
**Child testing characteristics**				
Child age at testing, mean (SD), y	4.8 (0.6)	4.9 (0.7)	4.9 (0.8)	.99
Year of testing				
1996-2005	21 (95.5)	37 (100)	36 (94.7)	.44
2006-2012	1 (4.5)	0	2 (5.3)	

Abbreviation: CES-D, Center for Epidemiological Studies Depression Scale; SSRI, selective serotonin reuptake inhibitor; NA, not applicable.

^a^ Differences between groups were calculated with χ^2^ and Kruskal-Wallis tests.

^b^ One patient in the not breastfed group was missing.

^c^ Includes American Indian or Alaska Native, Asian, and Native Hawaiian or Other Pacific Islander.

^d^ SSRI exposure during breastfeeding, n = 20; breastfed but no SSRI exposure, n = 35; not breastfed, n = 34.

^e^ Includes anxiety, bipolar disorder, and obsessive-compulsive disorder.

^f^ Includes bipolar disorder, bulimia nervosa, chemical imbalance, Epstein-Barr virus, grief, hypoglycemic symptoms, obsessive-compulsive disorder, postpartum depression, premenstrual syndrome, and stress.

^g^ SSRI exposure during breastfeeding, n = 20; breastfed but no SSRI exposure, n = 32; not breastfed, n = 32.

^h^ SSRI exposure during breastfeeding, n = 19; breastfed but no SSRI exposure, n = 31; not breastfed, n = 32.

^i^ SSRI exposure during breastfeeding, n = 16; breastfed but no SSRI exposure, n = 25.

### Statistical Analysis

First, distributions of the IQ scores were analyzed, and all scores and their residuals were determined to be approximately normally distributed. Baseline characteristics were compared between groups using the Pearson χ^2^ and Kruskal-Wallis tests, as appropriate. To study the potential association of the postnatal SSRI exposure with the child IQ scores separately from the association with breastfeeding, children exposed to SSRIs during breastfeeding were compared separately with breastfed children without postnatal SSRI exposure and nonbreastfed children. The crude results of both composite scores and scaled scores were measured as means (SDs) and compared between groups with an independent samples *t* test. The adjusted mean scores and their 95% CIs were analyzed with 1-way analysis of covariance adjusted for child age at testing, child sex, and prematurity (adjustment 1). In sensitivity analyses, the use of other psychotropic comedications and alcohol during pregnancy (adjustment 2) and duration of treatment with SSRIs during pregnancy and maternal CES-D scores during pregnancy (adjustment 3) were added to the model in a stepwise manner. The level of statistical significance was set as a 2-sided *P* < .05. In a secondary analysis, the Spearman correlation was used for exploring correlations between child IQ scores and maternal IQ scores and depressive symptoms. Cases with missing data on included covariates were excluded from adjusted analyses. Statistical analyses were performed with SPSS Statistics for Windows, version 29.0 (IBM Corporation).

## Results

### Participants

After exclusions for missing data on breastfeeding or postnatal SSRI exposure and prematurity, the final cohort consisted of 97 mother-child dyads (Figure 1). Of the 97 children, 52 (53.6%) were female and 45 (46.4%) male, and the mean (SD) age at cognitive testing was 4.9 (0.7) years. The mean (SD) maternal age at delivery was 34.0 (5.6) years. All 97 mothers identified as women. Eight women (8.2%) identified as Hispanic or Latina, 84 (86.6%) as non-Hispanic White, and 4 (4.1%) as other race or ethnicity, with 1 missing.

### Background Characteristics

The children were categorized into those exposed to SSRIs with breastfeeding (22 [22.7%]), breastfed children without postnatal SSRI exposure (37 [38.1%]), and nonbreastfed children, (38 [39.2%]). Eighty-one mothers (83.5%) were treated with fluoxetine hydrochloride. Other SSRIs included sertraline hydrochloride (9 [9.3%]), paroxetine (8 [8.2%]), and citalopram (2 [2.1%]). Three participants (3.1%) switched between SSRIs during pregnancy. The mean (SD) duration of treatment with SSRIs during pregnancy was 24.2 (15.6) weeks, with mothers who breastfed without SSRI treatment having a significantly shorter mean treatment duration than the 2 other groups (14.0 [12.4] weeks vs 35.0 [11.2] and 28.0 [14.9] weeks; *P* < .001) (Table 1). Twenty-five mothers (25.8%) were also treated with other psychotropic medications during pregnancy, including significantly fewer of the mothers who breastfed while treated with SSRIs (2 of 22 [9.1%]) than mothers who breastfed without SSRI treatment (8 of 37 [21.6%]) and mothers who did not breastfeed (15 of 38 [39.5%]) (*P* = .03) (Table 1). The other psychotropic medications used during pregnancy included benzodiazepines (16 of 97 [16.5%]), antipsychotics (5 of 97 [5.2%]), antiepileptic drugs (3 of 97 [3.1%]), tricyclic antidepressants (2 of 97 [2.1%]), lithium (2 of 97 [2.1%]), and buspirone hydrochloride (2 of 97 [2.1%]). During breastfeeding, 9 of 59 mothers (15.3%) reported any comedications, but none of these were other psychotropic medications. No participants reported any use of illicit drugs or cannabis during pregnancy, but 14 of 89 with data available (15.7%) reported use of tobacco and 48 of 89 (53.9%) reported use of alcohol at some point during pregnancy, with a significantly higher percentage of mothers who breastfed with and without SSRI use (13 of 22 [59.1%] and 26 of 37 [70.3%]) than mothers who did not breastfeed (9 of 38 [23.7%]) reporting alcohol use during pregnancy (Table 1). There were no differences between groups in maternal IQ, tested in a subset of 31 mothers (32.0%), or maternal depressive symptoms during pregnancy or at the time of developmental testing (Table 1).

Of the 97 children, 5 (5.2%) were born late preterm between gestational weeks 34 0/7 and 36 6/7. There was no significant difference in prematurity rates between exposure groups (Table 1). Seventeen children (17.5%) needed neonatal intensive care, without any significant differences among exposure groups. The mean (SD) documented duration of breastfeeding across the sample was 8.4 (8.2) months. Cognitive testing was performed with the WPPSI-R for 92 children (94.8%) and with the WPPSI-III for 5 children (5.2%).

### Composite Child IQ Scores

**Figure 2.**
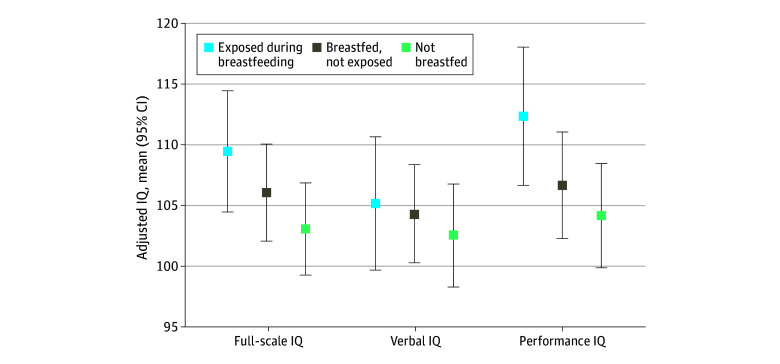
Adjusted Mean IQ Score of Children Exposed to Selective Serotonin Reuptake Inhibitors Through Breastfeeding, Breastfed Children Not Exposed Post Partum, and Nonbreastfed Children Adjusted means calculated between groups with 1-way analysis of covariance adjusted for child age at testing, child sex, and prematurity.

The mean (SD) FSIQ was 105.8 (11.8), corresponding to the 62nd percentile; VIQ, 103.9 (12.5), corresponding to the 58th percentile; and PIQ, 107.0 (13.0), corresponding to the 64th percentile. The adjusted mean FSIQ in children exposed to SSRIs through breastfeeding (109.4; 95% CI, 104.5-114.4) was similar to that in breastfed children not exposed to SSRIs through breastfeeding (106.1; 95% CI, 102.1-110.1; *P* = .29) and 6.3 points higher than in nonbreastfed children (103.1; 95% CI, 99.3-106.9; *P* = .046) (Table 2, Table 3, and Figure 2). The adjusted mean PIQ of the breastfeeding-exposed children was not significantly higher than of breastfed children not exposed to SSRIs post partum (112.3 [95% CI, 106.7-118.0] compared with 106.7 [95% CI, 102.3-111.1]; *P* = .12) but 8.1 points higher than the adjusted mean PIQ of nonbreastfed children (104.2; 95% CI, 99.9-108.5; *P* = .03). The adjusted mean VIQ scores were similar among all exposure groups. The differences in mean FSIQ and PIQ between the children exposed to SSRIs through breastfeeding and the nonbreastfed children were no longer statistically significant after further adjustments for factors related to maternal mood during pregnancy (Tables 2 and 3).

**Table 2.** Cognitive Performance of Breastfed Children With and Without Postnatal Exposure to SSRIs

WPPSI composite score	Participant group											
	SSRI exposure during breastfeeding (n = 22)				Breastfed but no SSRI exposure (n = 37)				*P* value between groups			
	Mean (SD)	Adjustment 1 mean (95% CI)^a^	Adjustment 2 mean (95% CI)^b^	Adjustment 3 mean (95% CI)^c^	Mean (SD)	Adjustment 1 mean (95% CI)	Adjustment 2 mean (95% CI)	Adjustment 3 mean (95% CI)	Crude^d^	Adjustment 1	Adjustment 2	Adjustment 3
FSIQ	109.4 (12.1)	109.6 (104.4-114.8)	109.3 (104.0-114.6)	112.0 (105.7-118.3)	106.2 (12.1)	106.1 (102.1-110.1)	106.5 (102.5-110.6)	104.3 (99.7-109.0)	.34	.29	.41	.08
VIQ	105.0 (12.9)	105.3 (100.0-110.6)	105.5 (100.2-110.9)	110.3 (103.9-116.6)	104.5 (12.2)	104.3 (100.3-108.4)	104.8 (100.6-108.9)	101.7 (97.0-106.3)	.89	.77	.82	.05
PIQ	112.4 (14.3)	112.5 (106.7-118.2)	111.6 (105.7-117.5)	110.9 (103.5-118.3)	106.8 (12.3)	106.7 (102.3-111.1)	107.1 (102.5-111.6)	106.5 (101.0-111.9)	.11	.12	.23	.38

Abbreviations: FSIQ, Full-Scale IQ; PIQ, Performance IQ; SSRI, selective serotonin reuptake inhibitor; VIQ, Verbal IQ; WPPSI, Wechsler Preschool and Primary Scales of Intelligence.

^a^ Calculated with analysis of covariance (ANCOVA) adjusted for child age at testing, child sex, and prematurity (adjustment 1).

^b^ Calculated with ANCOVA adjusted additionally for maternal psychotropic comedications and alcohol use during pregnancy (adjustment 2).

^c^ Calculated with ANCOVA adjusted additionally for duration of treatment with selective serotonin reuptake inhibitors during pregnancy and maternal depression scores during pregnancy (adjustment 3).

^d^ Calculated using an independent samples *t* test.

**Table 3.** Cognitive Performance of Breastfed Children Exposed to SSRIs and Nonbreastfed Children

WPPSI composite score	Participant group								*P* value between groups			
	SSRI exposure during breastfeeding (n = 22)				Not breastfed (n = 38)							
	Mean (SD)	Adjustment 1 mean (95% CI)^a^	Adjustment 2 mean (95% CI)^b^	Adjustment 3 mean (95% CI)^c^	Mean (SD)	Adjustment 1 mean (95% CI)	Adjustment 2 mean (95% CI)	Adjustment 3 mean (95% CI)	Crude^d^	Adjustment 1	Adjustment 2	Adjustment 3
FSIQ	109.4 (12.1)	109.4 (104.5-114.4)	109.0 (103.3-114.7)	108.3 (102.4-114.3)	103.1 (10.9)	103.1 (99.3-106.9)	103.4 (98.9-107.8)	105.6 (100.8-110.5)	.05	.046	.14	.52
VIQ	105.0 (12.9)	105.1 (99.5-110.7)	105.5 (99.0-111.9)	105.2 (98.4-112.0)	102.6 (12.7)	102.6 (98.3-106.8)	102.8 (97.8-107.8)	105.6 (100.2-111.0)	.49	.48	.54	.93
PIQ	112.4 (14.3)	112.3 (106.7-118.0)	112.0 (105.6-118.4)	110.8 (104.0-117.7)	104.2 (12.2)	104.2 (99.9-108.5)	103.9 (99.0-108.9)	105.1 (99.6-110.6)	.03	.03	.06	.23

Abbreviations: FSIQ, Full-Scale IQ; PIQ, Performance IQ; SSRI, selective serotonin reuptake inhibitor; VIQ, Verbal IQ; WPPSI, Wechsler Preschool and Primary Scales of Intelligence.

^a^ Calculated with analysis of covariance (ANCOVA) adjusted for child age at testing, child sex, and prematurity (adjustment 1).

^b^ Calculated with ANCOVA adjusted additionally for maternal psychotropic comedications and alcohol use during pregnancy (adjustment 2).

^c^ Calculated with ANCOVA adjusted additionally for duration of treatment with selective serotonin reuptake inhibitors during pregnancy and maternal depression scores during pregnancy (adjustment 3).

^d^ Calculated using an independent samples *t* test.

### Scores of Subtests

Comparisons of the scaled scores of the common subtests of WPPSI-R and WPPSI-III, as well as the subtests only included in WPPSI-R, are presented in eTables 1 and 2 in Supplement 1. All of the adjusted mean scores of the subtests across the groups were within 2 points of the standardized mean (SD) score of 10 (3) points, apart from Picture Completion. For Picture Completion, the adjusted mean scores were 1 SD above the standardized mean in all exposure groups (eTables 1 and 2 in Supplement 1). The adjusted mean results from 2 nonverbal subtests, Geometric Design and Mazes, were statistically significantly higher among children exposed to SSRIs during breastfeeding than nonbreastfed children, but the differences between adjusted group means were less than 2 points, and the adjusted mean scores of the remaining 10 subtests did not differ between exposure groups (eTable 2 in Supplement 1).

### Correlations

In the 83 children (85.6%) with available data on maternal CES-D scores, the FSIQ and VIQ scores were negatively correlated with the maternal estimated CES-D scores for the pregnancy period (*R* = −0.26 [*P* = .02] and *R* = −0.26 [*P* = .02], respectively). After stratification by exposure group, these correlation coefficients stayed constant but were no longer significant. There was no significant correlation between maternal CES-D scores during pregnancy and PIQ or between maternal CES-D scores at the time of neurodevelopmental testing and child IQ. In the 31 children (32.0%) with data available on maternal IQ, there was no significant correlation between maternal and child IQ.

## Discussion

In this cohort study of children born to mothers treated with SSRIs during pregnancy, the mean FSIQ, VIQ, and PIQ scores were similar between breastfed children with and without postnatal exposure to SSRIs. In the comparison between breastfed, postnatally SSRI-exposed children and nonbreastfed children, the FSIQ and PIQ scores of the breastfed, SSRI-exposed children were approximately one-half of an SD higher than the scores of their nonbreastfed counterparts. However, these differences were no longer significant after further adjustments for factors related to maternal mood during pregnancy. These results are supported by the comparison of the adjusted means of the subtests of WPPSI, which showed no significant differences between groups in the mean scores of the verbal subtests and slightly, but statistically significantly, higher mean scores of 2 of the nonverbal subtests in children exposed to SSRIs during breastfeeding than in nonbreastfed children.

The differences in FSIQ and PIQ scores between breastfed and nonbreastfed children in this SSRI-exposed cohort are of the same magnitude as the positive effects of breastfeeding seen on the cognitive development in the general population and in children exposed to maternal antiepileptic medications.^18,19^ This is, to our knowledge, a novel finding, supporting breastfeeding in mothers in need of treatment with SSRIs. However, the differences in FSIQ and PIQ between the breastfed SSRI-exposed and the nonbreastfed infants were no longer significant after adjustment for maternal factors, and there was a significant negative correlation between depressive symptoms during pregnancy and child IQ. Hence, these results also support the association between maternal mood disorders and the long-term development of the children described by previous studies.^28,29^

### Strengths and Limitations

The largest strength of this study was the standardized face-to-face testing of the exposed infants, which has previously been performed in only a limited number of studies on children prenatally exposed to SSRIs, and the differentiation on whether the children had been exposed during breastfeeding or not, which to our knowledge was unique.^2,4,30^ However, others have had a wider definition of cognitive performance and development, combining tests of cognitive performance, motor development, and parental questionnaires, or studied the relevant codes of *International Classification of Diseases* diagnoses in national registries.^1,2,31^ As these previous studies have not studied exposure during breastfeeding, our findings of the children exposed to SSRIs during breastfeeding performing as well as breastfed children without postnatal SSRI exposure are novel. These findings need to be confirmed by other studies with different outcome measures to acquire a more complete picture of the development of postnatally SSRI-exposed children.

This study also has some limitations. The secondary analysis of data previously collected for pregnancy studies was limited by the availability of breastfeeding data. Approximately one-third of the initial cohort was excluded due to missing data on breastfeeding and/or postpartum exposure to medications. After comparing the background characteristics and child IQs of these excluded mother-infant dyads with the rest of the cohort, we concluded that the missingness of breastfeeding questionnaires in these dyads was not differentially related to either the measured background characteristics or child IQ. Another limitation was that maternal depressive symptoms during pregnancy were measured retrospectively. Therefore, these scores were not included in the main analyses. Last, the cohort was based on volunteers and may not be representative of the general population. For example, all adjusted mean FSIQ, VIQ, and PIQ scores were above the average standardized means for the testing instrument. Therefore, caution should be used when comparing the IQ scores with those of other cohorts. However, as the background demographic characteristics were similar between exposure groups, there is support for the internal validity of the study. Larger and more diverse samples are needed to support the generalizability of these findings.

## Conclusions

In this prospective cohort study of children prenatally exposed to SSRIs, breastfed infants whose mothers were treated with SSRIs during breastfeeding had similar FSIQ, PIQ, and VIQ as breastfed infants who were SSRI exposed during pregnancy but not breastfeeding, and before adjustment for factors related to maternal mood during pregnancy, with somewhat higher FSIQ and PIQ scores than those of nonbreastfed children exposed to SSRIs during pregnancy. These findings are in line with previous studies that have shown positive effects of breastfeeding on child IQ. Based on these results, as well as other studies in the field, we conclude that mothers in need of treatment with SSRIs post partum may be encouraged to breastfeed without discontinuation of the treatment.

## Notes

Supplement 1. **eTable 1.** Scaled Scores of Verbal and Nonverbal Subtests of the Wechsler Test of Preschool and Primary Intelligence Compared Between Breastfed Children With and Without Postnatal Exposure to Selective Serotonin Reuptake Inhibitors **eTable 2.** Scaled Scores of Verbal and Nonverbal Subtests of the Wechsler Test of Preschool and Primary Intelligence Compared Between Breastfed Children Exposed to SSRIs Through Breastfeeding and Nonbreastfed Children

Supplement 2.
Data Sharing Statement

## References

[1] Suarez EA, Bateman BT, Hernández-Díaz S, . Association of antidepressant use during pregnancy with risk of neurodevelopmental disorders in children. JAMA Intern Med. 2022;182(11):1149-1160. 36190722 10.1001/jamainternmed.2022.4268 PMC9531086

[2] Galbally M, Lewis AJ, Buist A. Child developmental outcomes in preschool children following antidepressant exposure in pregnancy. Aust N Z J Psychiatry. 2015;49(7):642-650. 25698806 10.1177/0004867415569800

[3] Hermansen TK, Røysamb E, Augusti EM, Melinder A. Behavior and inhibitory control in children with prenatal exposure to antidepressants and medically untreated depression. Psychopharmacology (Berl). 2016;233(8):1523-1535. 26924747 10.1007/s00213-016-4248-3

[4] Nulman I, Koren G, Rovet J, . Neurodevelopment of children following prenatal exposure to venlafaxine, selective serotonin reuptake inhibitors, or untreated maternal depression. Am J Psychiatry. 2012;169(11):1165-1174. 23128923 10.1176/appi.ajp.2012.11111721

[5] Rommel AS, Bergink V, Liu X, Munk-Olsen T, Molenaar NM. Long-term effects of intrauterine exposure to antidepressants on physical, neurodevelopmental, and psychiatric outcomes: a systematic review. J Clin Psychiatry. 2020;81(3):19r12965. 32412703 10.4088/JCP.19r12965 PMC8739257

[6] Gorman JR, Kao K, Chambers CD. Breastfeeding among women exposed to antidepressants during pregnancy. J Hum Lact. 2012;28(2):181-188. 22344850 10.1177/0890334411429782

[7] Whaites Heinonen E, Johnson DL, Todd A, Chambers CD. Lower adherence to breastfeeding recommendations in mothers treated with antirheumatic and antidepressant medications. J Hum Lact. 2025;41(3):412-422. 40432295 10.1177/08903344251337384 PMC12238658

[8] Passier A, Woestenberg P. Chronic medication and breastfeeding, avoiding behavior in Dutch women. Neurotoxicol Teratol. 2023;98:107209.

[9] Zakaraya Z, Abu Assab M, Tamimi LN, . Pharmacokinetics and pharmacodynamics: a comprehensive analysis of the absorption, distribution, metabolism, and excretion of psychiatric drugs. Pharmaceuticals (Basel). 2024;17(3):280. 38543065 10.3390/ph17030280 PMC10974343

[10] Uguz F. A new safety scoring system for the use of psychotropic drugs during lactation. Am J Ther. 2021;28(1):e118-e126. 30601177 10.1097/MJT.0000000000000909

[11] Anderson PO. Drugs in lactation. Pharm Res. 2018;35(3):45. 29411152 10.1007/s11095-017-2287-z

[12] Weissman AM, Levy BT, Hartz AJ, . Pooled analysis of antidepressant levels in lactating mothers, breast milk, and nursing infants. Am J Psychiatry. 2004;161(6):1066-1078. 15169695 10.1176/appi.ajp.161.6.1066

[13] Sachs HC; Committee on Drugs. The transfer of drugs and therapeutics into human breast milk: an update on selected topics. Pediatrics. 2013;132(3):e796-e809. 23979084 10.1542/peds.2013-1985

[14] Hensch TK. Critical period plasticity in local cortical circuits. Nat Rev Neurosci. 2005;6(11):877-888. 16261181 10.1038/nrn1787

[15] Field T. Breastfeeding and antidepressants. Infant Behav Dev. 2008;31(3):481-487. 18272227 10.1016/j.infbeh.2007.12.004 PMC2556848

[16] Berle JØ, Steen VM, Aamo TO, Breilid H, Zahlsen K, Spigset O. Breastfeeding during maternal antidepressant treatment with serotonin reuptake inhibitors: infant exposure, clinical symptoms, and cytochrome p450 genotypes. J Clin Psychiatry. 2004;65(9):1228-1234. 15367050 10.4088/jcp.v65n0911

[17] Nordeng H, Bergsholm YK, Bøhler E, Spigset O. The transfer of selective serotonin reuptake inhibitors to human milk. Article in Norwegian. Tidsskr Nor Laegeforen. 2001;121(2):199-203. 11475200

[18] Bar S, Milanaik R, Adesman A. Long-term neurodevelopmental benefits of breastfeeding. Curr Opin Pediatr. 2016;28(4):559-566. 27386975 10.1097/MOP.0000000000000389

[19] Meador KJ, Baker GA, Browning N, ; Neurodevelopmental Effects of Antiepileptic Drugs (NEAD) Study Group. Breastfeeding in children of women taking antiepileptic drugs: cognitive outcomes at age 6 years. JAMA Pediatr. 2014;168(8):729-736. 24934501 10.1001/jamapediatrics.2014.118 PMC4122685

[20] Victora CG, Bahl R, Barros AJD, ; Lancet Breastfeeding Series Group. Breastfeeding in the 21st century: epidemiology, mechanisms, and lifelong effect. Lancet. 2016;387(10017):475-490. 26869575 10.1016/S0140-6736(15)01024-7

[21] Chambers CD, Braddock SR, Briggs GG, . Postmarketing surveillance for human teratogenicity: a model approach. Teratology. 2001;64(5):252-261. 11745831 10.1002/tera.1071

[22] Chambers CD, Johnson KA, Dick LM, Felix RJ, Jones KL. Birth outcomes in pregnant women taking fluoxetine. N Engl J Med. 1996;335(14):1010-1015. 8793924 10.1056/NEJM199610033351402

[23] Chambers CD, Johnson D, Xu R, ; OTIS Collaborative Research Group. Risks and safety of pandemic H1N1 influenza vaccine in pregnancy: birth defects, spontaneous abortion, preterm delivery, and small for gestational age infants. Vaccine. 2013;31(44):5026-5032. 24016809 10.1016/j.vaccine.2013.08.097

[24] Wechsler D. Wechsler Preschool and Primary Scale of Intelligence–Third Edition (WPPSI-III). APA PsycTests; 2002.

[25] Wechsler D. Wechsler Preschool and Primary Scale of Intelligence–Revised (WPPSI-R). APA PsycTests; 1989.

[26] Hollingshead AdB. Four Factor Index of Social Status. Yale University Department of Sociology; 1975.

[27] Radloff LS. The CES-D Scale: a self-report depression scale for research in the general population. Appl Psychol Meas. 1977;1(3):385-401. doi:10.1177/014662167700100306

[28] Gentile S. Untreated depression during pregnancy: short- and long-term effects in offspring—a systematic review. Neuroscience. 2017;342:154-166. doi:10.1016/j.neuroscience.2015.09.001 26343292

[29] Yin W, Pulakka A, Reichenberg A, . Association between parental psychiatric disorders and risk of offspring autism spectrum disorder: a Swedish and Finnish population-based cohort study. Lancet Reg Health Eur. 2024;40:100902. doi:10.1016/j.lanepe.2024.100902 38689608 PMC11059471

[30] Nulman I, Koren G, Rovet J, Barrera M, Streiner DL, Feldman BM. Neurodevelopment of children prenatally exposed to selective reuptake inhibitor antidepressants: Toronto sibling study. J Clin Psychiatry. 2015;76(7):e842-e847. doi:10.4088/JCP.14m09240 26231010

[31] Gentile S, Galbally M. Prenatal exposure to antidepressant medications and neurodevelopmental outcomes: a systematic review. J Affect Disord. 2011;128(1-2):1-9. doi:10.1016/j.jad.2010.02.125 20303599

