# Supplementary material for: Cognitive Outcomes of Children Exposed to Selective Serotonin Reuptake Inhibitors Through Breast Milk
Source: JAMA Netw Open. 2025 Nov 21;8(11):e2544989. doi: 10.1001/jamanetworkopen.2025.44989 (PMC12639480; doi:10.1001/jamanetworkopen.2025.44989)
Supplement: Supplement 1. — eTable 1. Scaled Scores of Verbal and Nonverbal Subtests of the Wechsler Test of Preschool and Primary Intelligence Compared Between Breastfed Children With and Without Postnatal Exposure to Selective Serotonin Reuptake Inhibitors eTable 2. Scaled Scores of Verbal and Nonverbal Subtests of the Wechsler Test of Preschool and Primary Intelligence Compared Between Breastfed Children Exposed to SSRIs Through Breastfeeding and Nonbreastfed Children [file jamanetwopen-e2544989-s001.pdf]

Supplementary Online Content

Heinonen EW, Kao K, Mattson SN, Chambers CD. Cognitive outcomes of children exposed to selective serotonin reuptake inhibitors through breast milk. *JAMA Netw Open.* 2025;8(11):e2544989. doi:10.1001/jamanetworkopen.2025.44989

eTable 1. Scaled Scores of Verbal and Nonverbal Subtests of the Wechsler Test of Preschool and Primary Intelligence Compared Between Breastfed Children With and Without Postnatal Exposure to Selective Serotonin Reuptake Inhibitors

eTable 2. Scaled Scores of Verbal and Nonverbal Subtests of the Wechsler Test of Preschool and Primary Intelligence Compared Between Breastfed Children Exposed to SSRIs Through Breastfeeding and Nonbreastfed Children

This supplementary material has been provided by the authors to give readers additional information about their work.

© 2025 Heinonen EW et al. *JAMA Network Open*.

eTable 1. Scaled Scores of Verbal and Nonverbal Subtests of the Wechsler Test of Preschool and Primary Intelligence Compared Between Breastfed Children With and Without Postnatal Exposure to Selective Serotonin Reuptake Inhibitors

WPPSI Scaled Score	SSRI exposed during breastfeeding n= 20		Breastfed but not exposed to SSRIs n= 35		p-value	
	**Mean (SD)**	**Adj. Mean[a] (95% CI)**	**Mean (SD)**	**Adj. Mean[a] (95% CI)**	**Crude[b]**	**Adj.**
**Verbal Subtests**						
Information	10.9 (2.4)	11.0 (10.0-11.9)	10.2 (1.9)	10.2 (9.4-10.9)	.34	.19
Comprehension	11.4 (2.7)	11.4 (10.3-12.4)	11.2 (2.2)	11.2 (10.4-12.0)	.94	.82
Vocabulary	11.0 (3.2)	10.9 (9.7-12.1)	11.7 (2.4)	11.8 (10.9-12.7)	.29	.27
Similarities	9.3 (1.9)	9.3 (8.3-10.3)	10.1 (2.2)	10.1 (9.3-10.8)	.15	.20
**Non-Verbal Subtests**						
Picture Completion	13.3 (2.3)	13.3 (12.1-14.5)	12.6 (2.8)	12.6 (11.7-13.5)	.47	.35
Object Assembly	10.8 (2.9)	10.7 (9.4-12.1)	11.0 (2.9)	11.1 (10.1-12.1)	.78	.71
Block Design	12.1 (2.3)	12.1 (11.1-13.1)	11.2 (2.1)	11.2 (10.5-12.0)	.33	.19
Sentences*	11.1 (2.6)	10.9 (9.6-12.1)	11.1 (2.5)	11.2 (10.3-12.1)	.94	.71
Arithmetic*	11.7 (2.4)	11.7 (10.3-13.1)	10.8 (2.9)	10.8 (9.8-11.8)	.43	.28
Animal Pegs*	10.4 (2.5)	10.2 (9.1-11.3)	11.0 (2.1)	11.1 (10.3-11.9)	.40	.22
Geometric Design*	10.9 (2.4)	11.0 (9.8-12.2)	9.8 (2.5)	9.8 (9.0-10.7)	.17	.13
Mazes*	11.4 (1.9)	11.5 (10.3-12.6)	10.5 (2.4)	10.5 (9.7-11.3)	.18	.16

Note: WPPSI=Wechsler Preschool and Primary Scales of Intelligence, Adj.= Adjusted, CI= Confidence Interval, IQ= Intelligence Quotient.

a: Adjusted mean composite scores of full-scale, verbal and performance intelligence quotient calculated with ANCOVA adjusted for child age at testing, child sex and prematurity (adj 1.),

b: Crude p-values calculated with Independent Samples T-Test.

*Only available for the 85 children who were tested with WPPSI-R.

© 2025 Heinonen EW et al. *JAMA Network Open*.

eTable 2. Scaled Scores of Verbal and Nonverbal Subtests of the Wechsler Test of Preschool and Primary Intelligence Compared Between Breastfed Children Exposed to SSRIs Through Breastfeeding and Nonbreastfed Children

WPPSI Scaled Score	SSRI exposed during breastfeeding n= 20		Not Breastfed n= 38		p -value	
	**Mean (SD)**	**Adj. Mean[a] (95% CI)**	**Mean (SD)**	**Adj. Mean[a] (95% CI)**	**Crude[b]**	**Adj.**
**Verbal Subtests**						
Information	10.9 (2.4)	10.9 (9.7-12.1)	10.3 (2.6)	10.3 (9.5-11.2)	.54	.45
Comprehension	11.4 (2.7)	11.4 (10.1-12.8)	10.3 (3.1)	10.3 (9.3-11.3)	.26	.18
Vocabulary	11.0 (3.2)	11.0 (9.7-12.4)	11.3 (3.0)	11.2 (10.2-12.2)	.69	.82
Similarities	9.3 (1.9)	9.2 (8.3-10.2)	9.8 (2.1)	9.8 (9.2-10.5)	.34	.30
**Non-Verbal Subtests**						
Picture Completion	13.3 (2.3)	13.3 (12.2-14.3)	12.8 (2.2)	12.8 (12.0-13.5)	.43	.43
Object Assembly	10.8 (2.9)	10.8 (9.6-12.0)	10.5 (2.7)	10.5 (9.6-11.4)	.60	.65
Block Design	12.1 (2.3)	12.1 (11.1-13.2)	10.8 (2.6)	10.8 (9.9-11.6)	.07	.06
Sentences*	11.1 (2.6)	11.0 (9.6-12.4)	9.5 (2.9)	9.5 (8.6-10.5)	.07	.09
Arithmetic*	11.7 (2.4)	11.6 (10.4-12.8)	10.3 (2.5)	10.3 (9.5-11.2)	.04	.09
Animal Pegs*	10.4 (2.5)	10.3 (9.2-11.4)	10.2 (2.2)	10.2 (9.4-11.0)	.66	.87
Geometric Design*	10.9 (2.4)	11.0 (9.8-12.1)	9.3 (2.2)	9.3 (8.5-10.1)	.02	.02
Mazes*	11.4 (1.9)	11.3 (10.2-12.4)	9.5 (2.5)	9.6 (8.8-10.3)	.01	.01

Note: WPPSI=Wechsler Preschool and Primary Scales of Intelligence, Adj.= Adjusted, CI= Confidence Interval, IQ= Intelligence Quotient.

a: Adjusted mean composite scores of full-scale, verbal and performance intelligence quotient calculated with ANCOVA adjusted for child age at testing, child sex and prematurity (adj 1.),

b: Crude p-values calculated with Independent Samples T-Test.

*Only available for the 85 children who were tested with WPPSI-R.

© 2025 Heinonen EW et al. *JAMA Network Open*.
