# Supplementary material for: Cognitive Outcomes of Children Exposed to Selective Serotonin Reuptake Inhibitors Through Breast Milk
Source: JAMA Netw Open. 2025 Nov 21;8(11):e2544989. doi: 10.1001/jamanetworkopen.2025.44989 (PMC12639480; doi:10.1001/jamanetworkopen.2025.44989)
Supplement: Supplement 2. — Data Sharing Statement [file jamanetwopen-e2544989-s002.pdf]

Data Sharing Statement

Heinonen. Cognitive Outcomes of Children Exposed to Selective Serotonin Reuptake Inhibitors Through Breast Milk. *JAMA Netw Open*. Published November 21, 2025.
doi:10.1001/jamanetworkopen.2025.44989

Data
Data available: Yes
Data types: Deidentified participant data
How to access data: Data supporting the findings will be available as per request to PI dr C. Chambers
When available: With publication

Supporting Documents
Document types: None

Additional Information
Who can access the data: Researchers whose proposed use of the data has been approved by the institutional review board.
Types of analyses: any purpose approved by the IRB
Mechanisms of data availability: after approval of proposal, as determined by PI dr Chambers
